# Removal of Pb from Contaminated Kaolin by Pulsed Electrochemical Treatment Coupled with a Permeable Reactive Barrier: Tuning Removal Efficiency and Energy Consumption

**DOI:** 10.3390/toxics11120961

**Published:** 2023-11-27

**Authors:** Yinyin Zhang, Libin Zang, Yuyan Zhao, Qiaoqiao Wei, Jiangtao Han

**Affiliations:** College of GeoExploration Science and Technology, Jilin University, Changchun 130026, China; zyyaaaaaa@163.com (Y.Z.); hanjt@jlu.edu.cn (J.H.)

**Keywords:** lead contamination, pulse electrochemical treatment, permeability reaction barrier, removal efficiency, energy consumption

## Abstract

Lead contamination in soil has emerged as a significant environmental concern. Recently, pulse electrochemical treatment (PECT) has garnered substantial attention as an effective method for mitigating lead ions in low-permeability soils. However, the impact of varying pulse time gradients, ranging from seconds to hours, under the same pulse duty cycle on lead removal efficiency (LRE) and energy consumption in PECT has not been thoroughly investigated. In this study, a novel, modified PECT method is proposed, which couples PECT with a permeable reaction barrier (PRB) and adds acetic acid to the catholyte. A comprehensive analysis of LRE and energy consumption is conducted by transforming pulse time. The results show that the LREs achieved in these experiments were as follows: PCb-3 s (89.5%), PCb-1 m (91%), PCb-30 m (92.9%), and PCb-6 h (91.9%). Importantly, these experiments resulted in significant reductions in energy consumption, with decreases of 68.5%, 64.9%, 51.8%, and 47.4% compared to constant voltage treatments, respectively. It was observed that LRE improved with an increase in both pulse duration and voltage gradient, albeit with a corresponding rise in energy consumption. The results also revealed that corn straw biochar as a PRB could enhance LRE by 6.1% while adsorbing migrating lead ions. Taken together, the present data highlights the potential of modified PECT technology for remediation of lead-contaminated soil, which provides an optimal approach to achieve high LRE while minimizing energy consumption.

## 1. Introduction

The accumulation of lead (Pb) contaminants on both the surface and within the soil matrix represents a significant threat to human health and the natural soil ecosystem [1]. Extensive research efforts have been devoted to addressing the remediation of soil contaminated with lead using physical, chemical, and biological approaches [2,3,4,5]. Nevertheless, these conventional techniques often entail prolonged operation, high energy consumption and significant labor. Consequently, there has been a growing exploration of low-energy, eco-friendly, and sustainable electrochemical treatment (ECT) technologies for remediating heavy metal–contaminated soils, especially those characterized by low-permeability properties [6]. Recently, ECT technology has achieved successful implementation at both laboratory and pilot scales for the removal of heavy metals from soil [7]. ECT technology is founded on three fundamental processes: electroosmosis, electromigration, and electrophoresis [4]. Upon the insertion of electrodes into the soil, an electric field is generated, which imparts energy to charged metal ions, compelling them to migrate in the opposite direction of their inherent charge, a phenomenon referred to as electromigration [8]. Electroosmosis pertains to the movement of pore water within the soil matrix’s porosity, induced by the presence of an electric double layer when an electric field is applied [9]. Electrophoresis, on the other hand, involves the motion of charged particles within an electric field [10]. The electrolysis reactions occurring at the anode and cathode lead to the generation of hydrogen ions and hydroxide ions [11]. During this electrolysis process, hydrogen ions are released and exchanged with positively charged metal ions located on the surface of soil particles, thereby facilitating the desorption and dissolution of metal ions within the vicinity of the anode [11]. Meanwhile, hydroxide ions produced at the cathode tend to accumulate and create complexes with heavy metal ions within the soil. This accumulation triggers precipitation and crystallization, thereby influencing the migration of metal ions [12].

In recent decades, ECT technology has emerged as a promising technique for remediating soil pollution [5,8,11,13,14,15,16]. Many recent advancements have been made in this field [17,18,19]. However, Ryu et al. confirmed that achieving a 75.7% removal efficiency for lead necessitates a treatment duration of 720 h and incurs an energy consumption of 1205 kWh/ton in the traditional ECT [14]. High removal efficiency and low energy consumption are major challenges for the field application of traditional ECT [20]. In order to balance the removal efficiency and associated costs, pulse electrochemical treatment (PECT) was modified from the traditional ECT [21,22]. Yuan et al. indicated that PECT technology involves intermittently controlling the flow of electric current at timed intervals, thereby periodically applying an electric field in the soil to drive the migration of heavy metal ions, achieving the removal of the latter [21,22]. Unlike traditional constant voltage treatment, PECT demonstrated clear advantages in reducing energy consumption and enhancing removal efficiency [23]. Ryu pointed out that pulse voltage could decrease energy consumption by adjusting power-off intervals while also reducing polarization effects and focusing phenomena, ultimately leading to improved removal efficiency [23]. Sun et al. achieved a significant reduction in energy consumption by applying a pulsed current of 0.2 mA/cm^2^ for the treatment of heavy metal-contaminated soil [24]. In a specific soil environment, the removal of heavy metals primarily relies on two factors: (1) the ability of electrolytic hydrogen ions to replace metal ions adsorbed on the soil surface and (2) the electromigration capability of metal ions in soil pores [25]. With regard to acid enhancers, numerous studies have substantiated that the inclusion of acidic enhancers could substantially enhance the removal efficiency of lead from soil [21,26,27]. Zhang et al. confirmed that removal efficiency increased by 20% in the ECT of Pb-contaminated kaolin by adding acetic acid [28]. Moreover, in recent years, various eco-friendly adsorbent materials have been employed in remediating heavy metal-contaminated soil by acting as permeable reactive barriers (PRBs) [29]. These materials exhibit strong adsorption capabilities, possess large specific surface areas, and contain abundant functional groups [19]. Biochar, as a superior PRB filling material, can be derived from a variety of organic and inorganic sources, including agricultural residues, forest residues, algal biomass, waste tires, and heavy crude oil [30]. He et al. utilized a new sheet PRB material, resulting in a significant reduction of residual heavy metals [26]. The comparison of lead removal efficiency and energy consumption under different electrochemical treatment conditions is displayed in Table S1 for ECT taking Pb and other heavy metals as an example.

Single ECT methods that can achieve an LRE of over 90% are rare [22], and there is even less research on PECT that simultaneously considers removal efficiency and electrical energy consumption for lead ion removal from soil [21,22]. Moreover, the concurrent application of various composite technologies often tends to prolong treatment time and increase voltage gradient, inevitably leading to an increase in time and electric energy costs [21,22]. Therefore, it is meaningful to explore a treatment technology that efficiently removes Pb from the soil while minimizing energy consumption. Yuan et al. employed a 12 h ON and 12 h OFF pulse treatment cycle, achieving an impressive removal efficiency of 93.5%. However, limited attention was given to the time gradient, and the 705 h treatment duration proved to be relatively lengthy, increasing time-related costs [22]. Based on previous research on the removal of Pb from the soil, a novel, modified PECT technology by coupling with PRB and adding acetic acid to catholyte was applied in this study. The varying pulse time gradients, ranging from seconds to hours, under the same pulse duty cycle were investigated. Zhou et al. showed that the pulse interval of 30 min ON/30 min OFF can achieve the highest removal efficiency for fluoride removal in soil [31]. Consequently, the effects of different pulse time gradients on heavy metal removal efficiency and energy consumption were compared by using a 1:2 pulse duty cycle. The pulse time gradient included seconds, minutes, and hours and consisted of four pulse interval periods: 3 s ON/3 s OFF, 1 min ON/1 min OFF, 30 min ON/30 min OFF, and 6 h ON/6 h OFF. In this study, corn straw biochar was utilized as a PRB and positioned adjacent to the catholyte compartment within the reactor. It exhibited a porous structure and a substantial specific surface area, enabling it to adsorb metal ions that were transported through pore water from the soil through electrostatic interactions, van der Waals forces, and capillary action [32]. Moreover, biochar contained numerous soluble ions within its structure, and its inherent carbonaceous nature served as a conductor for ions. Therefore, PRB not only adsorbed lead ions leached from the soil but also increased the electric current, playing a significant role in enhancing the removal efficiency [18,32].

This work focused on assessing the efficiency of lead removal from contaminated kaolin by using pulse voltage, with corn straw biochar serving as a PRB and acetic acid incorporated into the catholyte. The initial concentration of lead in the soil sample was 1153 mg kg^−1^. The primary objective was to conduct a comprehensive analysis of LRE and energy consumption by varying pulse time gradients in the PECT system coupled with the PRB. Furthermore, the study evaluated the potential of biochar in preventing secondary pollution in the catholyte and provided innovative design concepts for future remediation of lead-contaminated soil.

## 2. Materials and Methods

### 2.1. Chemicals and Materials

The kaolin sample used in this study was obtained from Lingshou County Dehang Mineral Products Inc. (Shijiazhuang, China); the calcination temperature of kaolin was between 700 °C and 1300 °C. The kaolin was calcined to a particle size of 200 mesh and artificially contaminated with Pb (CH_3_COO)_2_·3H_2_O solution at a concentration of 1 g L^−1^ of metal, with the soil moisture content maintained at 50%. The mixture of kaolin and solution was thoroughly stirred multiple times to ensure a homogeneous distribution of contaminants, and this process was continued for one month. The soil sample was then naturally dried and sequentially filtered using an 80-mesh sieve in a cool and dry laboratory environment. The initial concentration of Pb in the soil sample was determined to be 1153 mg kg^−1^. The soil pH was 5.77, and the electrical conductivity of the soil was 86.45 μs/cm, after mixing with lead acetate. The biochar used in this study was prepared from corn straw, which was purchased from Henan Lize Environmental Protection Technology Inc. (Shangqiu, China). The direct current (DC) power supplies were obtained from Chengde County Yuantao Trading Inc. (Chengde, China) and provided controllable DC voltage for the experiments. The relays were obtained from Leqing Lingyu E-commerce Inc. (Wenzhou, China). The electrochemical experimental reactor used in the study was manufactured by Hangzhou Zun-quan Acrylic Inc. (Hangzhou, China). The chemical properties of the initial samples and biochar are presented in Table 1 and Table 2.

### 2.2. Experimental Setup

**Figure 1:**
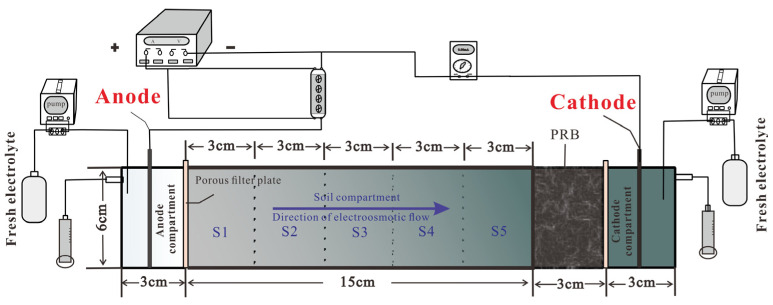
Experimental setup for pulse voltage power system.

A schematic of the electrochemical experimental reactor is shown in Figure 1. The reactor was constructed using plexiglass and had dimensions of 24 cm (length) × 4 cm (width) × 6 cm (depth). It consisted of four compartments: the soil compartment, two electrolytic compartments, and the biochar compartment. The corn straw biochar was utilized in the biochar compartment as a PRB and positioned adjacent to the catholyte compartment within the reactor. It can adsorb lead ions to prevent excessive heavy metals from entering the catholyte compartment [33]. The two electrolytic compartments received fresh electrolytes for the electrochemical progress and discharged the waste liquid at both ends of the electrolytic compartments. The biochar compartment was positioned adjacent to the soil compartments and the cathodic electrolytic compartment. The soil compartment refers to the specific area where the soil sample was placed and was divided into five equal sections (S1–S5) from the anode toward the cathode. A fiber filter paper was positioned in the partition layer of the compartment to facilitate filtration. To ensure a homogeneous distribution of the electric field, sheet-shaped graphite electrodes measuring 0.2 cm × 7 cm × 4 cm were inserted into the two electrolytic compartments. A wire connected in parallel to the longitudinal section of the compartment served to link the DC power source with the multimeter, allowing for the measurement of the electrical parameters. The relays were utilized to control the periodic ON/OFF function of the DC power. By regulating the ON/OFF time of the power, the relay facilitated the formation of a pulse-type ECT system in the soil. A multimeter was installed by wire between the DC power and the soil reaction device for monitoring the current. The study evaluated a total of nine experimental groups and compared the performance of different pulse voltage supply types, the presence of biochar-based PRB, voltage gradients, treatment durations, and interval periods of pulse voltage in ECT; the details of each experimental group are presented in Table 3.

### 2.3. Experimental Procedure

In this study, the soil samples were loaded into an experimental reactor designed for electrochemical experiments and were compacted layer by layer. Each layer of soil was compressed uniformly to minimize the presence of empty spaces between soil particles, ensuring consistent soil properties in the direction of the applied voltage. The compaction process continued until the soil surface was level with the drainage hole. To incorporate biochar, it was wrapped in filter paper and placed in the designated biochar compartment within the reactor. The anodic compartment was filled with deionized water while the cathodic compartment was filled with a 0.3 mol/L acetic acid solution. These electrolytes were intended to induce the desired electrochemical reactions and facilitate the electrochemical process within the experimental system. Graphite electrodes were used in the experimental setup and positioned in the respective electrolytic compartments [34,35]. To prevent direct contact between the electrodes and the soil samples, porous plexiglass plates and filter paper were employed as separation barriers. These materials allowed for the passage of electric current while maintaining a physical separation between the electrodes and the soil samples. This setup ensured the effective and safe application of the ECT to the soil samples. Before treatment, the soil samples were saturated with deionized water for 12 h. This step was conducted to ensure that the soil samples reached their maximum moisture capacity. By fully saturating the soil, the water content in the soil pores and voids was optimized.

All experiments were conducted at room temperature. The duration of the experiment was set to 72 h and 204 h, respectively. The soil compartment had a total length of 15 cm, with the biochar compartment and electrolytic compartments having a length of 5 cm. The experiment was designed to apply voltage gradients of 2 V/cm and 4 V/cm, corresponding to voltages of 42 V and 84 V, respectively. The experimental group employed a pulse voltage power supply system controlled by relays. Zhou et al. showed that the pulse interval of 30 min ON/30 min OFF achieved the highest removal efficiency for fluoride removal in soil [31], and Mu’azu et al. showed that heavy metal removal efficiency increased with the pulse duty cycle and voltage gradient increases [36]. Based on previous studies on the pulse duty cycle in the PECT system, the effects of different pulse time gradients on heavy metal removal efficiency and energy consumption were compared using a 1:2 pulse duty cycle. The pulse time gradient included seconds, minutes, and hours. It consisted of four interval periods of pulse voltage: 3 s ON/3 s OFF, 1 min ON/1 min OFF, 30 min ON/30 min OFF, and 6 h ON/6 h OFF. A peristaltic pump was used to introduce fresh electrolytes into the bottom of the electrolytic compartments. The electrolyte was regularly replenished to compensate for electrolytic and electroosmotic losses, which helped neutralize the accumulated anions in the electrolyte and regulate the pH of the electrolyte. Throughout the experiment, the pH of the anolyte and catholyte was measured every three hours. Current data from the multimeter were recorded, and the electroosmotic flow was measured every 12 h. The cathodic and anodic waste liquid was collected into storage bottles daily, and the concentration of Pb in the liquid was measured using an atomic absorbance spectrometer. After ECT, soil samples labeled S1, S2, S3, S4, and S5 were extracted. These soil samples were naturally dried for subsequent testing. The removal efficiency of lead in the soil was determined using Formula (1) [37]:

(1) $$R_{ave}=\frac{\sum_{k=1}^{n}{(C}_{ini}-C_{sk})/C_{ini}}{n}*100\%$$

where the concentration of the initial soil sample is $C_{ini}$; the concentrations of S1, S2, S3, S4, and S5 region are denoted as $C_{sk}$, with *k* = 1, 2, 3, 4 and 5; *n* is the number of sample points at the same distance to the anode and is 5 in this study. 

### 2.4. Instrumental Analysis

During the ECT process, all ECT experiments were carried out using a DC power (DP310, MAISITAIKE, Dongguan, China) supply to maintain a constant voltage gradient. The current intensity during the experiments was measured using a digital multimeter (DEM12, DELIXI, Wuhu, China). A multi-channel peristaltic pump (BT100-1L, LANGE, Shanghai, China) was used to supply fresh electrolytes to the electrolytic compartments at a steady flow rate of 505.4 µL/min. The evaluation of electroosmotic flow was conducted by connecting graduated cylinders to the cathodic compartment. The pH measurements of the soil samples and electrolyte were carried out at a temperature of 25 °C using a pH meter (PB-21, SARTORIUS, Gottingen, Germany). Before measurement, calibration of the pH meter with standard solutions was required. Following the standard for pH, 10 g of dry soil was blended with 25 milliliters of distilled water and agitated for 5 min. Subsequently, the suspension was allowed to settle for 30 min, and the pH of the supernatant solution was measured [38,39]. The total content of Pb in the soil samples was extracted using a microwave-assisted digestion system with a mixture of HNO_3_, HF, and HClO_4_, following the method described by Carignan and Tessier in 1988 [34]. The concentration of Pb within soil samples was measured by the X-ray Fluorescence Spectrometer (Soil handheld XRF Analyzer Explorer 9000, TIANRUI, Kunshan, China). In the measurement process, the soil samples need to undergo high-intensity pressing for 15 s using a bead machine. During measurement, it was crucial to ensure the stable placement of the instrument and verify that the probe had not been contaminated. The concentration of Pb ions in the liquid was measured by an atomic absorbance spectrometer (A3-AFG-12, PUXI, Beijing, China) [40]. When measuring the concentration of Pb in the solution, it was essential to preheat the instrument for 20 min. Afterward, the fume hood was opened, and the acetylene gas ignited. A visual inspection of the flame color was necessary to confirm its normalcy. Before measurement, calibration of the instrument was mandatory, and blank samples were set up. The preparation of standard samples should be accurate, ensuring reasonable concentration levels. Moisture content was determined through thermogravimetric analysis using an electric vacuum drying oven (GRX-12, SHANGHAIJINGHONG, Shanghai, China) for 24 h. The electrical conductivity of the soil samples was measured using a soil-to-water ratio of 1:2.5 and a calibrated conductivity meter (DDS-307A, REX, Shanghai, China) [40].

## 3. Results

### 3.1. Electrical Current

**Figure 2:**
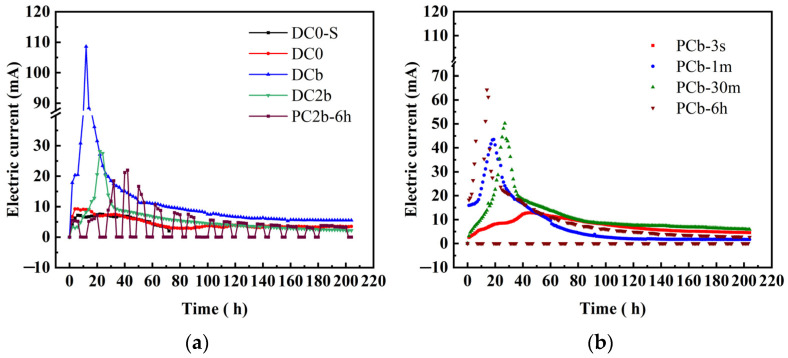
Electric current during electrochemical treatment (**a**,**b**).

Electrical current serves as an important indicator for assessing the movement of ions within soil pores [4]. All ECT experiments displayed a characteristic pattern of initially increasing and then decreasing electrical current. Figure 2a reveals that constant voltage coupled with biochar elicited a high current response. In DCb and DC2b, the peak value of electrical current appeared relatively early and was higher than in DC0 and PC2b-6 h. This suggested that the presence of multiple movable ions in biochar increased the overall number of ions capable of movement in the circuit, while the continuous and stable electric field generated by direct current accelerated ion movement in the soil. Especially for the DCb group, the current quickly reached a peak value in a short period, and the peak value was much higher than other groups. The DCb group used a voltage gradient of 4 V/cm with biochar. A high voltage gradient significantly increased the current density in soil pore water in a short period, and the conductive ions inherent in the biochar also increased the initial current density. A majority of ions in the soil and biochar quickly migrated to the cathodic region under the influence of a high field strength. This resulted in a reduction in the quantity of mobile ions in the soil pore water, leading to a significant downward trend in the current of the DCb group after 12 h [41]. The current stabilizes when the count of mobile ions remains constant. Figure 2b demonstrates that, initially, the current peak value exhibited the following order: PCb-6 h > PCb-30 m > PCb-1 m > PCb-3 s. A prolonged pulse time gradient enabled the continuous energy output for mobile ions over a specific duration, thereby activating the mobile ions adsorbed on the soil surface and biochar. The increase in the count of mobile ions results in an augmentation of current [2,25,42,43]. Lastly, the digital multimeter detected extremely small currents in the PCb-6 h group and PCb-30 m group during the power outage period. This phenomenon bore a similarity to the induced polarization method commonly employed in mineral exploration. The soil, characterized by its moisture content and the presence of heavy metals, can be considered a polarizable material. The entire soil matrix, in conjunction with the power source, effectively forms a closed electrical circuit. Notably, when a pulsed electric field was applied and subsequently turned off, a small electric current continued to flow within a certain range. This observation signified that when an electric current passed through, energy was stored within the soil medium. Typically, after the applied electric field was deactivated, this stored energy was released by maintaining the flow of electric current [44].

### 3.2. Electroosmotic Flow

In many studies, it has been observed that the electroosmotic flow (EOF) generally occurs from the anode to the cathode during treatment when the porous matrix has a negative surface charge and a low-intensity direct electric current is applied [45]. The principle of EOF was described by the Helmholtz–Smoluchowski theory. The electroosmotic flow rate $q_{eo}$ [m^3^/s] is calculated using Equation (2) [7,46]:

(2) $$q_{eo}=k_{eo}I\sigma$$

where $k_{eo}$ ((m^2^/s)/V) is the electroosmotic permeability coefficient, *I* is the electric current, and σ is the conductivity (Siemens/cm).

**Figure 3:**
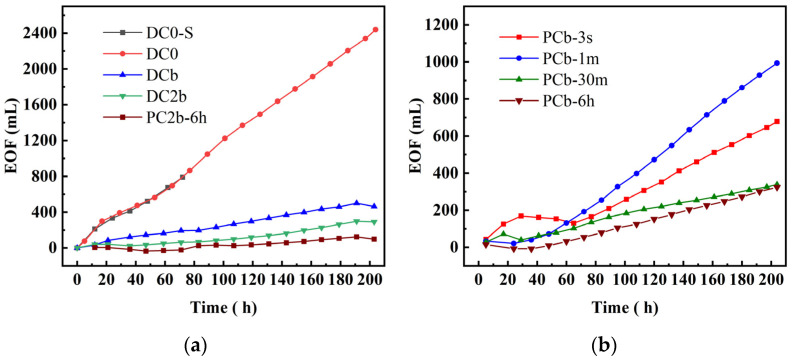
Electroosmotic flow during electrochemical treatment (**a**,**b**).

As shown in Figure 3a, the electroosmotic flow in DO0 exhibited a sharp increase, reaching a significantly high value of 2499 mL compared to other groups. The observed sequence of electroosmotic flow was DO0 > DCb > DC2b > PC2b-6 h, indicating that high electroosmotic flow could be generated under two conditions: a voltage gradient of 4 V/cm and a constant voltage power supply. In Figure 3b, during the initial stages, there was a slower rise in electroosmotic flow in the PCb-3 s group and PCb-30 m group compared to the other groups. This phenomenon can be inferred as a result of the rapid increase in current during the early stage, causing an acceleration in the electroosmotic flow. From 36 h to 65 h, the electroosmotic flow in the PCb-3 s group exhibited a small fluctuation, initially increasing and then decreasing. This was because the electroosmotic flow showed a reversal during the ECT process [47]. Based on the current trends, it could be inferred that the soil surface potential changed from negative to positive, which further contributed to the reversal of the electroosmotic flow under higher applied voltage [4,48]. Additionally, the intermittent interruption of the electric current during this period led to the cessation of electroosmotic flow. After 65 h, the electroosmotic flow gradually increased, and the electroosmotic flow rate followed the order of PCb-1 m > PCb-3 s > PCb-30 m > PCb-6 h. The underlying cause of this phenomenon can be explained by the trend in current distribution. When the current intensity was high, it led to reverse electroosmotic flow, which means that electroosmotic flow occurred from the cathode to the anode. However, the overall current trend consistently exhibited a prevailing anode-to-cathode flow tendency, which was more pronounced than the counterflow of electroosmotic fluid. Consequently, this caused a higher electroosmotic flow in the PCb-1 m group compared to the PCb-30 m and PCb-6 h groups. In contrast, the overall current intensity in the PCb-3 s group was weaker than that in the PCb-1 m group, resulting in a lower electroosmotic flow in the PCb-3 s group compared to the higher electroosmotic flow in the PCb-1 m group.

### 3.3. Moisture Content of Treated Soil

**Figure 4:**
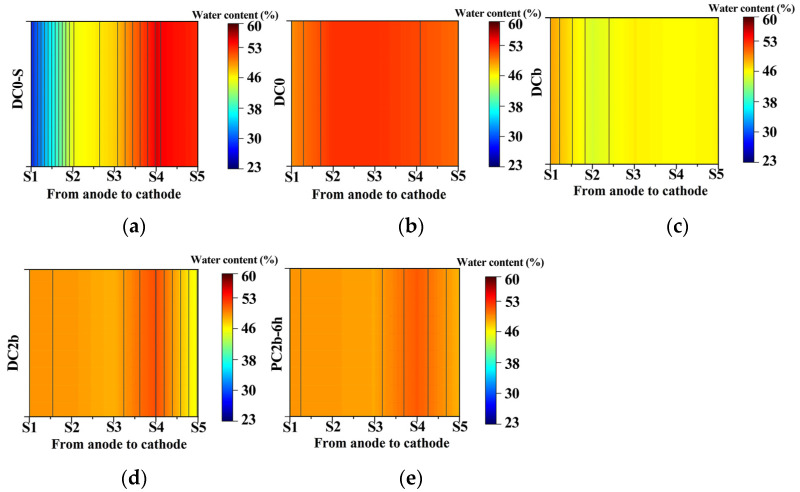
Moisture content of treated soil (**a**–**e**).

The moisture content of treated soil is a crucial factor in facilitating electromigration and electroosmosis, which are important mechanisms for the migration of heavy metals during the ECT process [49]. A previous survey indicated that the migration of heavy metals could occur when the soil moisture content in the treatment device exceeds 15% [48]. Changes in the moisture content of the treated soil in different regions of the experiment are shown in Figure 4a–e. The initial soil moisture content was 27.4%. In the presence of constant voltage power (DC0, DCb, DC2b) at 204 h, the variation in moisture content of treated soil was influenced by the processing time, voltage gradient, and the presence of biochar. Biochar has a water-buffering capacity and absorbs some water from adjacent soil areas. It also acts as a barrier to water molecules, limiting their penetration into the soil area. As a result, the soil moisture content of the DCb with biochar was lower than DC0. Furthermore, the generation of high electroosmotic flow, which was driven by a high voltage gradient, led to the transfer of moisture from the soil to the catholyte compartment. This was one of the reasons why the soil moisture content of DC2b was higher than that of DCb. The soil moisture content of DC0-S at 72 h of treatment time exhibited inhomogeneous distribution characteristics compared to DC0 due to the short processing time and incomplete physical and chemical reactions. Additionally, from the distribution of moisture content in the treated soil for DC2b and PC2b-6 h, it could be observed that the influence of the power supply type on the moisture content of treated soil was not significant under a voltage gradient of 2 V/cm. This phenomenon might be attributed to the comparatively lower voltage gradients in the DC2b and PC2b-6 h groups in comparison to the other groups. When the applied voltage was low, the electroosmotic flow rate within the soil pores tended to be more gradual, resulting in fewer concentration and polarization effects. Consequently, this led to a more consistent distribution of soil moisture content after treatment, with no significant variations.

### 3.4. Electrolyte pH

**Figure 5:**
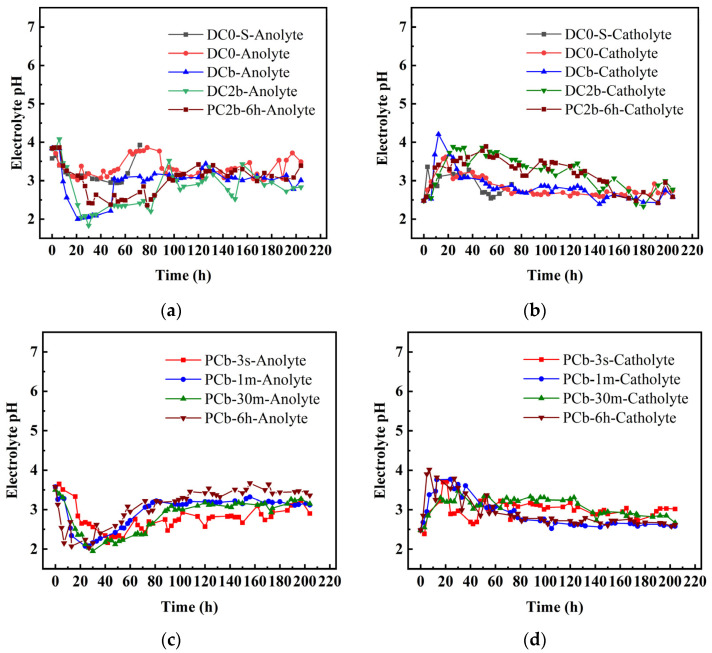
Electrolyte pH during electrochemical treatment (**a**–**d**).

Figure 5a–d illustrates the pH variation of the anolyte and catholyte. Numerous studies have investigated the impact of electrolyte pH during the ECT for treating contaminated soil [50]. Generally, in the absence of acidic additives for pH control, the electrolytic reaction in the ECT device leads to different levels of pH at each end of the soil compartment. The catholyte exhibits an alkaline pH, while the anolyte shows an acidic pH [51]. This difference in pH is caused by hydrolysis, which involves the generation of hydroxide ions at the cathode ($2H_{2}O+4e^{-}\to 2H_{2}\left(g\right)↑+4OH^{-}$) and hydrogen ions at the anode ($2H_{2}O-4e^{-}\to O_{2}\left(g\right)↑+4H^{+}$) [37]. Figure 5a,c presents the pH distribution of the anolyte over time for all electrochemical experiments. Initially, the pH of the anolyte decreased and then increased, ultimately stabilizing within a narrow range from 1.83 to 4.08. During the initial period of power on, the anolyte pH of the DCb, DC2b, PCb-1 m, PCb-30 m, and PCb-6 h experienced a significant decrease, which corresponded to the increased production of hydrogen ions when the current intensified. In Figure 5b,d, it could be observed that the pH of the catholyte of the DCb group and PCb-6 h group reached the highest value of pH at 12 h and 7 h, respectively, aligning with the enhanced production of hydroxide ions when the current substantially increased.

### 3.5. Soil pH and Soil Conductivity

**Figure 6:**
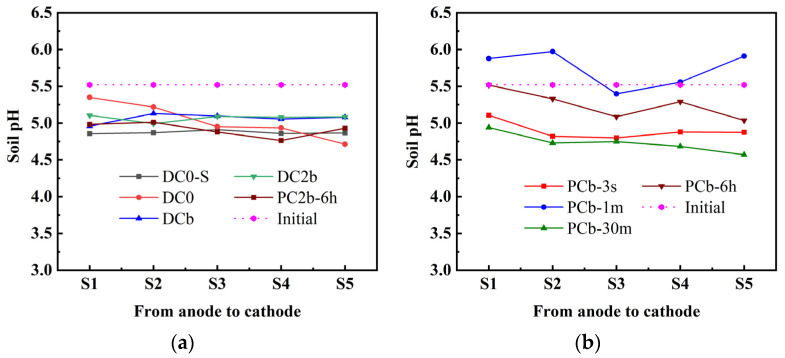
Soil pH of treated soil and initial soil pH (**a**,**b**).

The soil pH variation after ECT is depicted in Figure 6a,b. Initially, the soil pH of the Pb-contaminated soil was 5.77. After ECT progress, the soil samples were divided into five sections, labeled S1 to S5, based on their distance from the cathode. In Figure 6a, it can be observed that the pH of treated soil remained stable throughout the entire zone, ranging from 4.71 to 5.35. This stability was attributed to the adjustment of catholyte pH using a 0.3 mol/L acetic acid solution. The hydrogen ions provided by the acetic acid neutralize the hydroxide ions produced in the catholyte during electrolysis. Furthermore, the hydroxide ions generated in the middle of the soil combine with the hydrogen ions produced at the anode, resulting in a decrease in soil pH. This, in turn, led to a more uniform distribution of pH throughout the entire soil region. Figure 6b demonstrates the distribution of soil pH from S1 to S5 across the four experimental groups of pulse voltage. In the PCb-1 m and PCb-6 h treatment, the soil pH range fell between 5.04 and 5.97. In the PCb-3 s and PCb-30 m treatments, the soil pH range was between 4.51 and 5.11.

**Figure 7:**
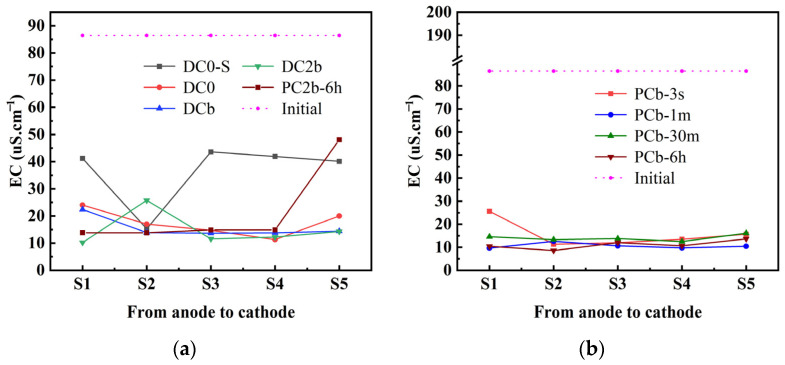
Conductivity of treated soil after electrochemical treatment (**a**,**b**).

In Figure 7a,b, the conductivity of soil after the ECT is illustrated. The initial conductivity of the soil was 86.45 μs/cm. Except for the PCb-6 h treatment, which had a high value in the S5 region, the distribution of electrical conductivity in the treated soil was homogeneous. In the experiments of pulse voltage at 204 h, the electrical conductivity ranged from 8.6 μs/cm to 25.75 μs/cm. The conductivity of the soil was influenced by the presence of conductive ions. The addition of acetic acid as the catholyte neutralizes hydroxide ions in the soil through diffusion, promoting the desorption of soluble ions that were adsorbed on the soil surface [4,40,52]. These ions then migrate to different soil areas under the influence of the electric field, resulting in a decrease in conductive ions in the soil. However, there were significant differences in the distribution of electrical conductivity in the treated soil of the DC0-S. Based on these observations, it could be concluded that the short electric treatment time affected the chemical and physical reactions of various substances in the soil. When the treatment time was short, these reactions could be incomplete, leading to inconsistencies in the distribution pattern of electrical conductivity across different soil regions.

### 3.6. Residual Content of Pb in Collected Catholyte

**Figure 8:**
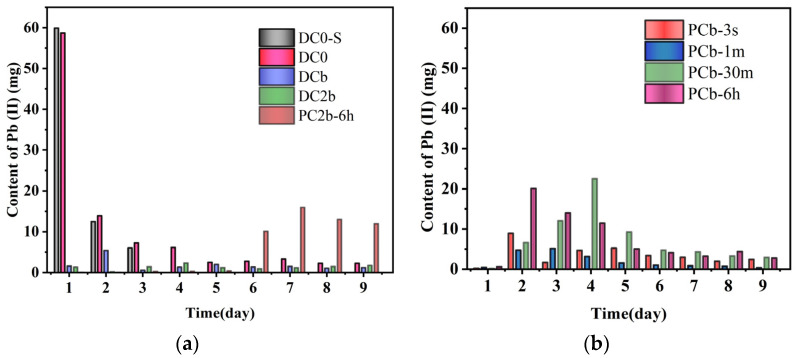
Residual Pb ion content (mg) in collected catholyte over time (day) (**a**,**b**).

Since Pb ions primarily migrate to the cathode through electromigration and electroosmotic flow, the monitoring of Pb ions was carried out in the catholyte, while the content of Pb ions in the waste liquid discharged from the anolyte compartment was not detected. Figure 8a illustrated the trends in the content of Pb ions in the catholyte during the treatment of constant voltage. Initially, a substantial number of Pb ions were discharged from the soil on the first day, followed by a slow migration in the DC0-S and DC0 treatment. On the other hand, the voltage gradient had a slightly positive effect, with the content of Pb ions in the catholyte being higher under a voltage gradient of 4 V/cm compared to 2 V/cm during the early stages of the experiment. In the pulse voltage systems, the trends in the content of Pb ions in the catholyte discharge liquid differed from those in the constant voltage systems. The initial content of Pb ions in the catholyte was low, and only trace amounts were discharged after the third day in the PC2b-6 h group. Figure 8b demonstrates that the PCb-6 h group reached the peak of Pb ion release two days earlier than the PCb-30 m group. However, the overall trend was to increase first and then decrease. The results revealed that the PRB had a notable and negative effect on the cumulative content of Pb ions in the catholyte. The biochar material utilized in the PRB effectively adsorbed Pb ions, impeding their migration from the PRB compartment to the catholyte [53,54]. These findings highlighted the significant impact of the PRB on the migration of Pb ions from the PRB compartment to the catholyte compartment. The presence of the PRB impeded the Pb ions movement, causing them to accumulate in the PRB and be gradually released over time. Furthermore, the voltage gradient also played a role in influencing the content of Pb ions in the catholyte. The specific mechanisms behind these observations require further analysis and investigation.

### 3.7. Removal Efficiency of Pb

**Figure 9:**
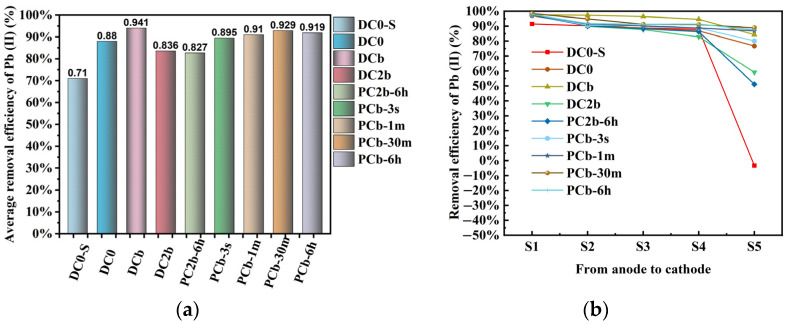
The average removal efficiency (%) of Pb in soil and the removal efficiency (%) of Pb in each soil region (**a**,**b**).

The removal efficiency of Pb is affected by various experimental conditions [7,47,55,56]. The experiment investigated five variable conditions, including the type of power supply (constant voltage or pulse voltage), the presence of a PRB made of biochar, different voltage gradients, different treatment times, and different interval periods of pulse voltage. The experimental results in Figure 9a present the average removal efficiency of Pb from soil. Firstly, it was observed that the average removal efficiency of Pb in the continuously energized group with constant voltage (DC2b) and the group subjected to pulse voltage (PC2b-6 h) with a 6 h interval period was essentially the same. The mechanism of Pb migration facilitated by biochar as a PRB was analyzed by comparing the features observed in DC0 and DCb. In general, biochar not only adsorbed migrated Pb ions but also enhanced the current intensity in the soil circuits. This increased current intensity promoted the migrated rate of Pb, resulting in a higher removal efficiency for the DCb group compared to the DC0 group. Furthermore, when biochar was combined with a voltage gradient of 4 V/cm, the DCb group exhibited higher removal efficiency for Pb compared to the DC2b group, which operated under a voltage gradient of 2 V/cm. Previous research has shown that long-term treatment is beneficial for improving the removal efficiency of heavy metals [57]. In this study, the removal efficiency of DC0 was 17% higher than that of the corresponding group with short treatment times (DC0-S). Furthermore, the removal efficiency of Pb was remarkably high at a higher voltage gradient of 4 V/cm and a treatment time of 204 h, using biochar as the PRB in the DCb, PCb-3 s, PCb-1 m, PCb-30 m, and PCb-6 h groups. The corresponding removal efficiency for these groups was 94.1%, 89.5%, 91%, 92.9%, and 91.9%, respectively. There were differences in the average removal efficiency among the PCb-3 s, PCb-1 m, PCb-30 m, and PCb-6 h groups in the pulse voltage system. Specifically, the average removal efficiency of Pb in the PCb-1 m, PCb-30 m, and PCb-6 h groups was 1.5%, 3.4%, and 2.4% higher, respectively, than that of the PCb-3 s group.

The removal efficiency of Pb in each soil region from S1 to S5 is presented in Figure 9b. In the S1 region of soil, the removal efficiency of Pb exceeded 97% in the groups at a treatment time of 204 h. The removal efficiencies of the nine experimental groups from S1 to S5, were high, surpassing 80%. Notably, the removal efficiency of the S5 region was a crucial factor that influenced the average removal efficiency. In the S5 region of the DC0-S groups, there was a noticeable inflection point, attributed to the difference in ECT duration. Most experimental groups underwent a 204 h ECT, whereas the DC0-S group had a shorter treatment duration of 72 h. Unlike the other experimental groups, applying a high voltage gradient over a shorter treatment period caused mobile charged ions within the soil to move in the opposite direction of the electrical current within the soil pores. Specifically, positively charged lead ions migrated toward the cathode direction, leading to their gradual accumulation in the S5 region. However, the shorter treatment duration was not conducive to the lead ions in the S5 region migrating into the catholyte compartment, resulting in a significant accumulation of lead ion within the S5 region. This observation suggests that the system of pulse voltage was effective in desorbing heavy metal ions from the soil surface; the brief power-off period in the system of pulse voltage reduced the polarization effect in the soil [4,38,58,59]. Consequently, it allowed the acid ions from the catholyte to gradually diffuse toward the middle of the soil within the fluid present in the soil pores. This diffusion process was beneficial for decreasing the alkalinity in the portion of the soil middle and facilitated the desorption and migration of lead, which was adsorbed on the soil surface, towards the catholyte during the subsequent cycle of the electric field.

### 3.8. Electrical Energy Consumption

The electrical energy consumption can be calculated based on the applied current, voltage, and cross-sectional area of the soil in the vertical current direction. The calculation formula is as follows [60]:

(3) $$E=\frac{1}{V_{s}}\int UI\;dt$$

where *E* is the energy consumption per unit volume of soil (kWh/m^3^), *V_s_* represents the soil volume (m^3^), U represents the voltage (V), *I* represents the current (A), and *t* represents the treatment time (h).

**Figure 10:**
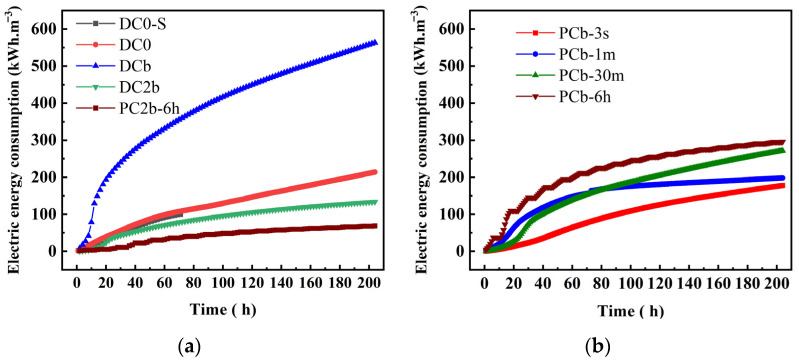
Electric energy consumption during electrochemical treatment (**a**,**b**).

The energy consumption and lead removal efficiency in all experiment groups are presented in Table 4. The electrical energy consumption during ECT using corn straw biochar as a PRB system is shown in Figure 10a,b. In Figure 10a, the energy consumption of the DC0-S, DC0, DCb, DC2b, and PC2b-6 h groups was 99.6 kWh/ m^3^, 213.9 kWh/m^3^, 563.2 kWh/m^3^, 133.5 kWh/m^3^, and 68.4 kWh/m^3^, respectively. The DCb group was significantly higher than the other experiments. This was likely due to the high electric current observed in the group with a high voltage gradient and biochar used as PRB under constant voltage. This indicated that a high voltage gradient and treatment time were important factors, contributing to high energy consumption. It is noteworthy that in Figure 10a, the PC2b-6 h group exhibited lower energy consumption compared to the DC2b group. The result suggests that the interval of power outage in the experiment reduced the effective treatment time, which was beneficial for reducing energy consumption. As shown in Figure 10b, the energy consumption of PCb-6 h, PCb-30 m, PCb-1 m, and PCb-3 s was 296 kWh/m^3^, 271.6 kWh/m^3^, 197.9 kWh/m^3^, and 177.6 kWh/m^3^. The order of energy consumption in the experimental group was as follows: PCb-6 h > PCb-30 m > PCb-1 m > PCb-3 s. Compared to the traditional experiment (DCb) using a constant voltage coupled with PRB for treatment of Pb-contaminated soil, the groups using pulse voltage power, including PCb-3 s, PCb-1 m, PCb-30 m, and PCb-6 h, reduced energy consumption by 68.5%, 64.9%, 51.8%, and 47.4%, respectively.

The utilization of the pulse voltage involved integrating different electrodynamic enhancement technologies [61,62,63,64], such as the addition of acetic acid in the catholyte, the establishment of a PRB, and the application of a voltage gradient of 4 V/cm. These approaches collectively aimed to reduce the polarization effects that typically occur in the traditional ECT [13,40,43,59]. Through this process, they enhanced the outcomes in Pb removal by stimulating the desorption of Pb from the soil surface. Moreover, these methods aided in reducing the electrical conductivity of soil.

## 4. Conclusions

This study confirmed the high efficiency of the PECT system coupled with the PRB in lead removal from soil. The LREs were found to be 89.5% (PCb-3 s), 91% (PCb-1 m), 92.9% (PCb-30 m), and 91.9% (PCb-6 h), with particularly remarkable results in the S1 region, where removal efficiency reached 97%. Furthermore, under the same pulse duty cycle conditions, shorter pulse time gradient in seconds and minutes (3 s ON/3 s OFF and 1 min ON/1 min OFF) demonstrated lower current requirements during the ECT process, resulting in reducing energy consumption. Specifically, energy consumption in the experiments for PCb-3 s, PCb-1 m, PCb-30 m, and PCb-6 h was 177.6 kWh/m^3^, 197.9 kWh/m^3^, 271.6 kWh/m^3^, and 296 kWh/m^3^, which represented a significant reduction when compared to the constant voltage coupled with PRB experiment (DCb), where energy consumption reached as high as 563.2 kWh/m^3^ under some voltage gradients. Consequently, the energy consumption was reduced, with percentage decreases of 68.5%, 64.9%, 51.8%, and 47.4%, respectively.

In contrast to previous PECT methods for treating lead-contaminated soil, the present research revealed that the PECT system coupled with PRB, designed with shorter pulse time gradients, effectively achieved high LRE while significantly reducing energy consumption and shortening the treatment duration. Additionally, concerning the treatment of secondary pollution introduced by the catholyte, the study highlighted the effective adsorption capabilities of corn straw biochar. The temporal evolution of lead content in the catholyte indicated that the optimal time to replace biochar falls within the first days of pulse treatment.

In summary, the present data provided an innovative ECT method for lead-contaminated soil, offering high removal efficiency, lower time requirements, and energy savings. However, further research is necessary to investigate the optimal strategies for remediating different soil types and using modified biochar to achieve maximal adsorption of lead ions from the catholyte.

## Figures and Tables

**Table 1:** Initial chemical properties of kaolin.

The Properties of Kaolin	Value
pH	5.77 ± 0.21
EC (μs/cm)	86.45 ± 25.74
SiO_2_ (%)	54.42 ± 0.11
Al_2_O_3_ (%)	42.68 ± 0.09
TiO_2_ (%)	1.77 ± 0.01
Fe_2_O_3_ (%)	0.58 ± 0.009
CaO (%)	0.23 ± 0.007
K_2_O (%)	0.13 ± 0.005
P_2_O_5_ (%)	0.08 ± 0.002
MgO (%)	0.06 ± 0.001
SrO (%)	0.03 ± 0.001
Cr_2_O_3_ (%)	0.02 ± 0.001

**Table 2:** Initial chemical properties of biochar.

The Properties of Biochar	Value
Organic carbon content (%)	42.21 ± 0.21
Total nitrogen content (%)	8.34 ± 0.05
Total phosphorus content (%)	2.31 ± 0.01
Total potassium content (%)	16.12 ± 0.09
Ash content (%)	7.23 ± 0.03
Others (%)	23.79 ± 0.11
pH	9.46 ± 0.05

**Table 3:** Experimental parameters of the ECT programs.

No.	PRB	Power Type	Power On/Off Interval Periods	Voltage Gradient (V/cm)	Treatment Time (h)
DC0-S	no PRB	DC power	Constant voltage	4	72
DC0	no PRB	DC power	Constant voltage	4	204
DCb	biochar	DC power	Constant voltage	4	204
DC2b	biochar	DC power	Constant voltage	2	204
PCb-3 s	biochar	PC power	3 s/3 s	4	204
PCb-1 m	biochar	PC power	1 min /1 min	4	204
PCb-30 m	biochar	PC power	30 min/30 min	4	204
PCb-6 h	biochar	PC power	6 h/6 h	4	204
PC2b-6 h	biochar	PC power	6 h/6 h	2	204

**Table 4:** The removal efficiency of Pb (%) and energy consumption in all experiment groups.

Exp.	S1 (%)	S2 (%)	S3 (%)	S4 (%)	S5 (%)	Average Removal (%)	Energy Consumption (kWh/m^3^)
DC0-S	91.5	90.2	88.9	87.9	−3.4	71	99.6
DC0	97	90.9	88.6	86.9	76.6	88	213.9
DCb	97.6	97.4	96.5	94.6	84.4	94.1	563.2
DC2b	98.1	89.9	87.8	82.8	59.2	83.6	133.5
PCb-3 s	97.6	90.6	90.4	88.8	80.2	89.5	177.6
PCb-1 m	97.4	91.6	90.1	88.8	86.9	91	197.9
PCb-30 m	98.4	94.9	91.3	91.1	88.9	92.9	271.6
PCb-6 h	97.9	91.7	91.3	91.4	87.5	91.9	296
PC2b-6 h	97.4	90.2	88.3	86.4	51.1	82.7	68.4

The soil sample region was delineated by the distance from the anode toward the cathode, designated as S1, S2, S3, S4, and S5.

## Data Availability

Some or all data, models, or codes that support the findings of this study are available from the corresponding author upon reasonable request.

## Supplementary Materials

The following supporting information can be downloaded at: https://www.mdpi.com/article/10.3390/toxics11120961/s1, Table S1: The comparison of lead removal efficiency and energy consumption under different electrochemical treatment conditions.
