# Supplementary material for: Removal of Pb from Contaminated Kaolin by Pulsed Electrochemical Treatment Coupled with a Permeable Reactive Barrier: Tuning Removal Efficiency and Energy Consumption
Source: Toxics. 2023 Nov 27;11(12):961. doi: 10.3390/toxics11120961 (PMC10747039; doi:10.3390/toxics11120961)
Supplement: Supplementary file 1 [file toxics-11-00961-s001.zip › toxics-2697233-supplementary.pdf]

Table S1. The comparison of lead removal efficiency and energy consumption under different electrochemical treatment conditions

Soil type	Research method	Catholyte enhancer	Anolyte enhancer	Pulse interval	Initial Pb concentration	Treatment time	Removal efficiency (%)	Energy consumption	Reference
Mixed Clay Soils	PECT	HNO_3	NaOH	9 h ON/1 h OFF	100 mg/kg	504 h	17 (Pb) 62.41 (Cu)	1151.58 kwh/m^3	(Usman et al., 2012) [1]
Kaolin	PECT	0.2 mol/L Citric acid 0.05 mol/L $Cacl_2$	0.2 mol/L Citric acid 0.05 mol/L $Cacl_2$	12 h ON/12 h OFF	240.8 mg/kg	705 h	93.5 (Pb)	124.8 kWh/m^3	(Yuan et al., 2017) [2]
Farm soil	AACE with EDTA leaching	EDTA	EDTA	Constant voltage	10000 ppm (Cu) 1000 ppm (Pb) 100 ppm (Cd)	6 h	71.2 (Cu) 95.3 (Pb) 98.8 (Cd)	Not mentioned	(Xu et al., 2019) [3]
Paddy soil	ECT	0.1 N HNO_3	0.1N NaOH	Constant voltage	298.7 mg/kg	720 h	60.1 (Cu) 75.7 (Pb) 43.1 (As)	1204.6 kWh/ton	(Ryua et al., 2011) [4]
Farmland soil	ECT with anode approaching	0.1 mol/L KNO_3	0.1 mol/L KNO_3	Constant voltage	853.3 mg/kg	219 h	31.3 (Pb) 25.93 (Cd)	227 kWh/t	(Zhang et al., 2016) [5]
Calcareous soil	ECT	0.01 mol/L EDTA	0.01 mol/L EDTA	Constant voltage	250 mg/kg	120 h	40.11 (Zn) 43.10 (Cd) 24.7 (Pb)	Not mentioned	(Beyrami 2021) [6]
Kaolin	ECT	0.5 N H_2SO_4	0.005N H_2SO_4	Constant voltage	1438 mg/kg	96 h	49.8 (Pb)	Not mentioned	(Kim et al., 2001) [7]
Kaolin	ECT	0.1 N EDTA	0.1N EDTA	Constant voltage	1167.9 mg/kg	10 h	65.1% (Pb)	78.3 kWh/m^3	(Taneja et al., 2023) [8]
Kaolin	ECT	EDTA-2Na and acetic acid	EDTA-2Na and acetic acid	Constant voltage	500 mg/kg	96 h	76.4% (Pb)	Not mentioned	(Liu et al., 2023) [9]
Kaolin	ECT with PRB	2 M KNO_3	2 M KNO_3	Constant voltage	296.5 ± 6.3 mg/kg	192 h	62.2 ± 2.5% (Pb)	Not mentioned	(Liu et al., 2022) [10]

Kaolin	ECT with PRB	Not mentioned	Not mentioned	Constant voltage	1000 mg/kg	120 h	92.6% (Pb)	Not mentioned	(Zhou et al., 2021) [11]
Kaolin	PECT with PRB	0.3mol/L acetic acid	distilled water	3 s ON/3s OFF 1min ON/1min OFF 30 min ON/30min OFF 6 h ON/6h OFF	1153 mg/kg	204 h	89.5%)PCb-1m (91%) PCb-30m (92.9%) PCb-6h (91.9%) (Pb)	177.6 kWh/m^3 197.9 kWh/m^3 271.6 kWh/m^3 296 kWh/m^3	Present study

ECT: Electrochemical treatment; PECT: Pulse electrochemical treatment; AACE: Asymmetrical alternating current electrochemistry

References

1. Usman, A.-K.; Mu'azu, N.-D.; Lukman, S.; Essa, M.; Bukhari, A.A.; Al-Malack, M.H.Removal of Lead and Copper from Contaminated Mixed Clay Soils Using Pulsed Electrokinetics. Soil Sediment Contam. *Int. J.* **2020**, *29*, 465–480. https://doi.org/10.1080/15320383.2020.1743969.
2. Yuan, L.; Xu, X.; Li, H.; Wang, Q.; Wang, N.; Yu, H. The influence of macroelements on energy consumption during periodic power electrokinetic remediation of heavy metals contaminated black soil. *Electrochim. Acta.* **2017**, *235*, 604–612. https://doi.org/10.1016/j.electacta.2017.03.142.
3. Xu, J.; Liu, C.; Hsu, P.-C.; Zhao, J.; Wu, T.; Tang, J.; Liu, K.; Cui, Y. Remediation of heavy metal contaminated soil by asymmetrical alternating current electrochemistry. *Nat. Commun.* **2019**, *10(1)*, 2440. https://doi.org/10.1038/s41467-019-10472-x.
4. Ryu, B.G.; Park, G.-Y.; Yang, J.-W.; Baek, K. Electrolyte conditioning for electrokinetic remediation of As, Cu, and Pb-contaminated soil. *Sep. Purif. Technol.* **2011**, *79*, 170–176. https://doi.org/10.1016/j.seppur.2011.02.025.
5. Zhang, P.; Jin, C.; Sun, Z.; Huang, G.; She, Z. Assessment of Acid Enhancement Schemes for Electrokinetic Remediation of Cd/Pb Contaminated Soil. *WATER AIR SOIL POLL.* **2016**, *227(6)*, 217. https://doi.org/10.1007/s11270-016-2879-y.
6. Beyrami, H. Effect of different treatments on electrokinetic remediation of Zn, Pb and Cd from a contaminated calcareous soil. *Chin. J. Chem. Eng.* **2021**, *38*: p. 255-265. https://doi.org/10.1016/j.cjche.2020.09.011.
7. Kim, S.; Moon, S.-H.; Kim, K.-W. Removal of Heavy Metals from Soils using Enhanced Electrokinetic Soil Processing. *WATER AIR SOIL POLL.* **2001**, *125*: p. 259–272. https://doi.org/10.1023/A:1005283001877.
8. Taneja, S.; Karaca, O.; Haritash, A.-K. Treatment of Pb-contaminated soil by electrokinetics: Enhancements by varying voltage, chelant, and electrode material. *J. Geochem. Explor.* **2023**, *250,*107240. https://doi.org/10.1016/j.gexplo.2023.107240.
9. Liu, Y.; Zhuang, Y.-F.; Xiao, F.; Liu, Z. Mechanism for reverse electroosmotic flow and its impact on electrokinetic remediation of lead-contaminated kaolin. *Acta Geotech.* **2023**, *18*, 124885. https://doi.org/10.1007/s11440-022-01640-3.
10. Liu, Q.; Zhang , Q.; Jiang, S.; Du, Z.; Zhang, X.; Chen, H.; Cao, W.; Nghiem, L.-D.; Ngo, H.-H. Enhancement of lead removal from soil by in-situ release of dissolved organic matters from biochar in electrokinetic remediation. *J. Cleaner Prod.* **2022**, *361*, 132294. https://doi.org/10.1016/j.jclepro.2022.132294.
11. Zhou, H.; Liu, Z.; Li, X.; Xu, J. Remediation of lead (II)-contaminated soil using electrokinetics assisted by permeable reactive barrier with different filling materials. *J. Hazard. Mater.* **2021**, *408*, 124885. https://doi.org/10.1016/j.jhazmat.2020.124885.
